# Spatiotemporal Regulation of Transcript Isoform Expression in the Hippocampus

**DOI:** 10.3389/fnmol.2021.694234

**Published:** 2021-07-08

**Authors:** Joun Park, Shannon Farris

**Affiliations:** ^1^Fralin Biomedical Research Institute, Center for Neurobiology Research, Virginia Tech Carilion, Roanoke, VA, United States; ^2^Department of Biomedical Sciences and Pathobiology, Virginia-Maryland College of Veterinary Medicine, Virginia Tech, Blacksburg, VA, United States; ^3^Virginia Tech Carilion School of Medicine, Roanoke, VA, United States

**Keywords:** RNA localization, alternative splicing, subcellular localization, hippocampus, Cdc42, BDNF, alternative isoform expression, alternate UTR

## Abstract

Proper development and plasticity of hippocampal neurons require specific RNA isoforms to be expressed in the right place at the right time. Precise spatiotemporal transcript regulation requires the incorporation of essential regulatory RNA sequences into expressed isoforms. In this review, we describe several RNA processing strategies utilized by hippocampal neurons to regulate the spatiotemporal expression of genes critical to development and plasticity. The works described here demonstrate how the hippocampus is an ideal investigative model for uncovering alternate isoform-specific mechanisms that restrict the expression of transcripts in space and time.

## Introduction

**Figure 1:**
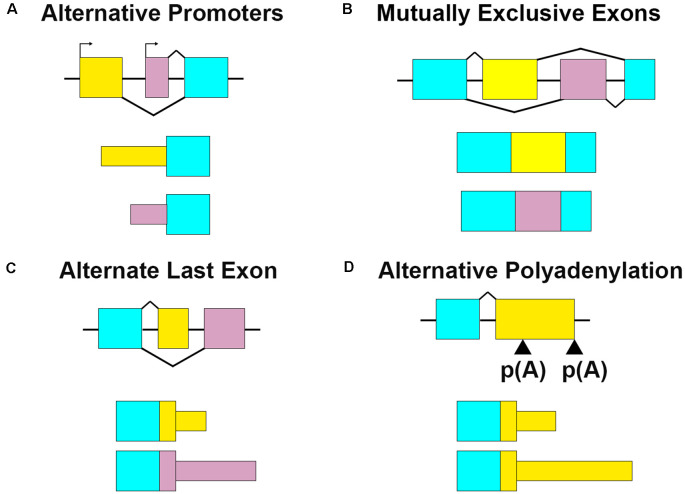
Multiple RNA processing mechanisms contribute to mRNA sequence variation. **(A)** Alternative promoters change the 5′ ends of transcripts by initiating transcription from different start sites. For *Bdnf*, the alternate promoter determines its dendritic localization. **(B)** Mutually exclusive exons retain one exon or the other. Gria2 uses this mechanism in combination with RNA editing to modify RNA localization and channel characteristics. **(C)** Alternate last exons retain one or the other mutually exclusive terminal exons. Cdc42 uses two alternate last exons to vary the terminal coding and 3′UTR sequences, which affect transcript localization and protein properties. **(D)** Alternative polyadenylation (APA) terminates transcription at multiple places within the same last exon. Also termed tandem 3′UTR, this results in a shortened or elongated 3′UTR. Camk2a harbors multiple 3′UTR lengths through this process, which modifies its posttranscriptional regulatory capacity.

The transcription and translation of RNA must be meticulously organized for cells to function properly. Neurons, in particular, are morphologically complex cells that require gene expression to be regulated in specific subcellular compartments at specific times for proper development and function. As a result, many different forms of RNA regulation coordinate to ensure proper transcript expression in the right place at the right time. Every step of RNA metabolism, from trafficking to translation and degradation is dictated in an mRNA-specific fashion by the nucleotide sequence and the combination of factors that associate with it, otherwise known as the RNA regulation code (Moore, 2005; Raj and Blencowe, 2015). Alternative isoform expression is one regulatory mechanism well poised to mediate spatial and temporal gene expression in neurons. Alternative isoform expression results from the interplay of many different RNA regulatory processes, including alternative promoter usage (alternative first exon; Twine et al., 2013), alternative exon splicing (Ha et al., 2021; Joglekar et al., 2021), alternative last exon usage (Taliaferro et al., 2016), and alternative polyadenylation (APA; Fontes et al., 2017; Ha et al., 2021; Figure 1). This coordinated inclusion or exclusion of specific *cis* RNA sequences can determine the spatiotemporal expression profile of a given transcript *via* differential binding of *trans*-acting factors. These include RNA binding proteins (RBPs) and microRNAs, that then recruit other post-transcriptional regulators, such as motor proteins or translational regulators (Wang et al., 2009; Mayya and Duchaine, 2019; Yee et al., 2019). Alternative isoform expression is regulated by a complex interplay of splicing factors (Fischer et al., 2011; Carey and Wickramasinghe, 2018), epigenetic modifications (Zhang et al., 2020), transcription factors (Thompson et al., 2019), enhancers/repressors (Conboy, 2021), and RNA binding proteins (Yee et al., 2019). Neuron-enriched RBPs, such as RBFOX1 (Jacko et al., 2018; Begg et al., 2020), ELAVL (Hinman et al., 2013; Yokoi et al., 2017), NOVA (Jensen et al., 2000; Ule et al., 2006), and MBNL2 (Wang et al., 2012, 2015; Taliaferro et al., 2016), regulate neuronal-specific, or even neuron class-specific (Feng et al., 2021), splicing programs by binding to highly conserved sequence motifs in pre-mRNAs and recruiting spliceosome factors to promote or inhibit splicing of specific exons.

Alternative isoform expression greatly expands the diversity of RNA transcripts and can be used to change transcriptome profiles at different developmental stages (Su et al., 2018). Over 95% of human multi-exon genes are alternatively spliced, and alternative splicing occurs at a higher rate in the brain than other tissues, demonstrating the importance of RNA variation in neurons (Yeo et al., 2004; Pan et al., 2008). Temporally-controlled isoform expression is critical for shaping neuron function across development by impacting ion channel composition (Gray et al., 2007), intracellular junction formation (Grabowski and Black, 2001; Iijima et al., 2016; Südhof, 2017), plasticity-related protein localization (Hermey et al., 2017; Furlanis and Scheiffele, 2018), and microRNA-mediated control over local translation (Hu and Li, 2017; Corradi and Baudet, 2020). Uncovering the mechanisms and impact of alternative isoform regulation on the spatiotemporal organization of gene expression is crucial to our understanding of neuronal biology.

RNA localization is a process by which transcripts are transported to different areas of the cell where they can be locally translated. This is especially important in neurons, where functionally distinct compartments demand specialized gene expression at discrete time points (Steward and Schuman, 2003). RNA localization is indispensable for compartment-specific proteomes, as almost half of all neurite-localized proteins are translated locally (Zappulo et al., 2017). Certain alternative isoform expression modes, such as alternative 3′ untranslated region (UTR) usage, can drive RNA localization. Over 70% of neuron-enriched genes have at least two alternative 3′UTRs, and longer 3′UTRs are concentrated in neurites over soma, demonstrating their importance in RNA trafficking (Ouwenga et al., 2017; Tushev et al., 2018). Longer 3′UTR sequences, acquired by alternative last exon usage or APA, allow for a greater number of regulatory sequence motifs to control the localization, stability, and translation transcripts (Andreassi and Riccio, 2009). Other RNA regulatory mechanisms, such as RNA editing and microRNA processing, work in tandem with alternative isoform expression to facilitate accurate localized gene expression and local translation. RNA editing can mediate localization through RNA sequence changes, and microRNAs can repress target RNAs in a spatially restricted manner (Kumar and Carmichael, 1997; Zhang and Carmichael, 2001; Schratt, 2009).

The hippocampus serves as an ideal model for studying RNA regulation dynamics because its principal neurons are physically separated into distinct cell bodies and dendritic layers, allowing for easy identification of synaptically localized RNAs. Due to its high propensity for plasticity, the hippocampus requires constant changes in gene expression to meet and maintain synaptic demands. In response to synaptic activity, hippocampal synapses readily undergo plasticity to strengthen or weaken connections in processes called Long-term potentiation (LTP) and Long-term depression (LTD), respectively (Bliss and Lomo, 1973; Dudek and Bear, 1992; Neves et al., 2008). The local translation of newly transcribed and/or synaptically localized transcripts is required to maintain activity-dependent changes to synaptic efficacy (Frey and Morris, 1997; Nguyen and Kandel, 1997; Holt et al., 2019). The hippocampus is also an area of robust splicing. It is estimated that every gene expresses an average of 3.9 alternative splice isoforms in the rat hippocampus (Wang et al., 2019). Our lab identified 3,298 differentially spliced transcript isoforms from 2,111 unique genes across the mouse hippocampal subregions and compartments, showing how splicing and localization are prevalent in this region (Farris et al., 2019). In this review, we cover several compelling examples in which splicing and RNA localization aid in hippocampal development and plasticity (Table 1). We further highlight recent work cataloging the extent of compartment-specific isoform regulation in the hippocampus and underscore the field’s need for mechanistic studies to reveal how alternate isoforms and their interactors functionally impact local translation.

**Table 1:** Summary of transcript isoforms discussed in this review.

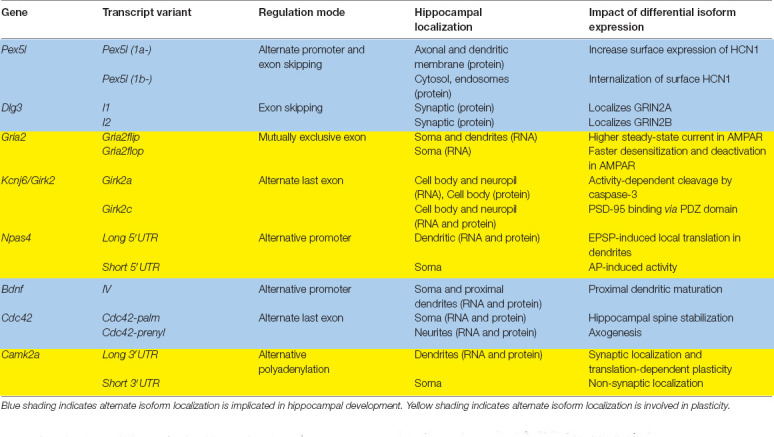

## Developmentally-Regulated Isoform Expression Guides Ion Channel Function

Splicing regulation of ion channels is critical to shaping the electrical activity required for hippocampal development (Grabowski and Black, 2001; Lipscombe, 2005). Mislocalization or mistiming of ion channel expression has been linked to neurodevelopmental diseases such as cortical malformations and epilepsies (Smith and Walsh, 2020). Thus, the investigation of how developing hippocampal neurons use RNA regulation to manage ion channel expression is an actively investigated topic. Here we discuss two examples of how splicing can be used to ensure the proper localization of ion channels during critical developmental timepoints.

The localization of hyperpolarization-activated cyclic nucleotide-gated 1 (HCN1), a channel responsible for the I_h_ current that modulates action potential firing frequency, is mediated by the splicing of peroxisomal biogenesis factor 5 like (*Pex5l*; Santoro et al., 2004). Neuronal *Pex5l* is an HCN1 channel subunit chaperone transcribed from two alternate promoters, 1a and 1b (Lewis et al., 2009). The nomenclature for the 11 *Pex5l* splice variants uses a parenthesis after *Pex5l* to indicate which N-terminal exons are spliced to the common exons 5–16 (Han et al., 2020). PEX5L variants containing exon 1b appear to oppose exon 1a-containing variants by decreasing HCN1 expression and I_h_ current through increased endocytosis of surface HCN1 channels. Specifically, PEX5L (1b-2–4) reduces HCN1 expression, and (1b-2) results in the sequestering of HCN1 to intracellular puncta in hippocampal CA1 (Lewis et al., 2009; Santoro et al., 2009). These two (1b−) variants make up almost all of the PEX5L (1b−) variants in the mouse brain, with their mRNA accounting for 20–30% of all *Pex5l* mRNA (Santoro et al., 2009). Independently, PEX5L (1a) prevents the axonal expression of HCN1, while PEX5L (1a-4) localizes HCN1 to the distal dendritic layer of CA1 (Lewis et al., 2009; Santoro et al., 2009; Piskorowski et al., 2011). While all *Pex5l* transcripts containing the 1a exon are highly expressed in the rat hippocampus from P2 to adulthood, the expression of isoforms containing exon 1b appears later, around P13 (Lewis et al., 2009). This suggests developmental regulation of the alternate exon 1b promoter in the hippocampus. Indeed, indicators of transcriptional activation (histone-3 lysine 4 trimethylation, H3K4me3) cluster almost exclusively to the exon 1a promotor in neural progenitors (Meissner et al., 2008; Lipscombe and Pan, 2009). In whole brain lysates, there are H3K4me3 peaks at both exon 1a and 1b, showing activation of the 1b promoter later in development. This early inhibition of the I_h_ -reducing exon 1b may facilitate the role of I_h_ in generating giant depolarizing potentials in the initial postnatal period that are critical for synaptic strengthening (Bender et al., 2001, 2005; Kasyanov et al., 2004) or as a pacemaker for early network oscillation (Garaschuk et al., 1998). The oscillations mediated by HCN channels may provide a synchronous activity that is essential to the hippocampal maturation of glutamatergic neurotransmission (Durand et al., 1996). Thus, restriction of exon 1b-containing PEX5L variant expression during early development allows for proper hippocampal activity and maturation through the localization and expression of HCN1.

Like PEX5L, alternative splicing of discs large MAGUK scaffold protein 3 (*Dlg3*, also known as synapse-associated protein 102 or *Sap102*) regulates the localization of ion channel subunits during development, specifically glutamate ionotropic N-methyl-d-aspartate receptors (NMDARs) that mediate synaptic plasticity (Chen et al., 2011). During development, there is an NMDAR subunit composition switch from NMDA type subunit 2B (GRIN2B) to 2A (GRIN2A), leading to an increase in the activation threshold for LTP (Monyer et al., 1994; Watanabe et al., 1994). This switch is mediated by *Dlg3*, a member of the PSD-95-like membrane-associated guanylate kinase family of synaptic scaffolding proteins (Müller et al., 1996; Smith et al., 1996). DLG3 expression in CA1 synapses closely mirrors that of GRIN2B at CA1 during development, and DLG3 has been found to control GRIN2B expression in the synapse (Sans et al., 2000; Chen et al., 2012). Mammalian *Dlg3* has three isoforms which can be differentiated based on the presence or absence of two alternatively spliced regions (Müller et al., 1996). The I1 region at the N terminus binds GRIN2A, whereas the I2 region found towards the C-terminal end binds GRIN2B and localizes it to synapses (Chen et al., 2011; Wei et al., 2015, 2018). Knockdown of *Dlg3* I1 in hippocampal neurons increases GRIN2B surface expression, and knockdown of *Dlg3* I2 increases surface expression of GRIN2A (Chen et al., 2012; Wei et al., 2015). In mice, the expression of *Dlg3* mRNA containing I1 increases from P1 to P20 (Chen et al., 2011). Conversely, *Dlg3* mRNA containing I2 decreases relative to the other variants from P2 to P20, and I2 phosphorylation, which clusters DLG3 at spines, plateaus after P8 (Chen et al., 2011; Wei et al., 2015, 2018). Thus, developmentally regulated splicing from I2 containing- to I1 containing*-Dlg3* aids in the stage-specific subunit composition switch of GRIN2B to GRIN2A containing NMDARs at maturing hippocampal synapses. In this way, hippocampal neurons developmentally tune activity patterns *via* the splicing regulation of ion channel chaperones.

## Multiple RNA Processing Mechanisms Contribute to Proper Ion Channel Subunit Expression

As illustrated with *Pex5l* and *Dlg3*, splicing serves as an important regulator of hippocampal channel localization. Controlling the flow of ions is the basis for neuronal signaling, and making sure the correct ion channels localize to their appropriate compartments is crucial to hippocampal function. One strategy to accomplish this is by directing channel subunit isoforms to specific areas to regulate both the channel localization and properties. Several different posttranscriptional processing modes are utilized to achieve this, including alternative last exons, mutually exclusive splicing, and RNA editing. *Kcnj6* and *Gria2* demonstrate how channel subunit splicing can affect localization, and how RNA editing adds another layer to this regulation.

Potassium inwardly-rectifying channel subfamily J member 6 (*Kcnj6*) expresses splice variants whose RNAs localize differently. Known more widely as G protein-gated inwardly rectifying K+ channel 2 (*Girk2*), it is a G-protein dependent hyperpolarizing potassium channel that maintains resting membrane potentials and has been shown to depotentiate LTP in hippocampal cultures (Sakura et al., 1995; Chung et al., 2009; Hibino et al., 2010). Two *Girk2* splice isoforms, GIRK2a and GIRK2c, differ due to alternate last exons, resulting in different 3′UTRs and 11 amino acids at the C-terminus (Wei et al., 1998). Although both mRNA variants are expressed in the CA1 neuropil, *Girk2c* RNA and protein have a much higher expression (Marron Fernandez de Velasco et al., 2017). In addition, GIRK2a protein is mostly restricted to the cell body of hippocampal pyramidal neurons (Marron Fernandez de Velasco et al., 2017). GIRK2c’s PDZ binding motif allows its binding to several synaptic proteins that GIRK2a cannot, such as PSD-95 (Inanobe et al., 1999). Experiments with *Girk2* knockout mouse hippocampal slices indicate that GIRK2a mediates slow inhibitory postsynaptic currents in CA1 proximal dendritic fields *via* Schaeffer collaterals, while GIRK2c mediates them in the distal dendritic fields *via* perforant/temporoammonic path (Marron Fernandez de Velasco et al., 2017). Furthermore, caspase-3 cleaves GIRK2a at the alternatively spliced C-terminus after prolonged activity in rat hippocampal cultures (Baculis et al., 2017). This cleavage decreases binding to its G-protein Gβγ and GIRK1, preventing GIRK2’s activity and surface expression. Thus, splicing of GIRK2 to the *Girk2c* variant localizes the RNA and protein to the neuropil, and only GIRK2a is cleaved after sustained activity.

Another channel subunit gene, glutamate ionotropic receptor AMPA type subunit 2 (*Gria2*), can undergo two modes of RNA regulation that affect its activity and localization- splicing and editing (Tanaka et al., 2000; Barbon and Barlati, 2011). GRIA2 is a subunit of alpha-amino-3-hydroxy-5-methyl-4-isoxazole-propionic acid receptors (AMPARs) that mediate fast excitatory synaptic transmission (Lüscher et al., 1999; Tanaka et al., 2000). Insertion and recycling of AMPARs, and thus GRIA2, mediates changes in synaptic strength in response to learning (Lüscher et al., 1999; Ehlers, 2000; Zheng et al., 2015). RNA editing is a process through which protein sequences are altered *via* nucleoside modification of target transcripts. It is estimated that 85% of all human pre-mRNAs undergo the most common form of RNA editing, A-to-I editing (Athanasiadis et al., 2004). This editing results in the chemical conversion of an adenosine to inosine, which is read as a guanosine (Bass and Weintraub, 1987, 1988; Wagner et al., 1989; Walkley and Li, 2017). *Gria2* undergoes A-to-I RNA editing at two positions known as the Q/R site and the R/G site. Q/R site editing changes the amino acid at position 607 from glutamine to arginine (Higuchi et al., 1993; Yang et al., 1995). *Gria2* that is unedited at the Q/R site is readily trafficked to the membrane, while the edited form is sequestered in the ER (Araki et al., 2010; Gurung et al., 2018). A mutation of *Gria2* such that it can no longer be edited at position 607 results in loss of neurons in CA1 (Konen et al., 2020). Q/R editing makes AMPAR less permeable to Ca^2+^ (Egebjerg and Heinemann, 1993). *Gria2* mRNA is also edited at the R/G site to change an arginine in position 764 to glycine (Lomeli et al., 1994). This has a drastic effect on its localization, as 56% of *Gria2* mRNA in the rat hippocampus is edited at the R/G site, while only 12% of *Gria2* mRNA is R/G edited in the synaptosomal fraction (La Via et al., 2013). The R/G edit increases the recovery rate of AMPAR from desensitization (Lomeli et al., 1994).

*Gria2* undergoes alternative splicing at the mutually exclusive “flip/flop” exons near the R/G site (Sommer et al., 1990). GRIA2 variants containing the flop exon (GRIA2Flop) have a faster desensitization rate, faster deactivation rate, and lower steady-state current than those containing the GRIA2Flip variant (Wen et al., 2017). *Gria2flip* accounts for 41% of total *Gria2* mRNA in rat hippocampus, with the number rising to 96% in the synaptoneurosomal fraction (La Via et al., 2013). In the rat CA1, *Gria2flip* mRNA localizes to the cell soma (stratum pyramidale) and dendritic (stratum radiatum) layers, while *Gria2flop* mRNA is expressed exclusively in the cell soma (La Via et al., 2013). Blocking sodium or calcium currents in rodent CA1 with tetrodotoxin or nifedipine, respectively, results in upregulation of *Gria2flop* RNA (Sommer et al., 1990; Penn et al., 2012). While flip/flop splicing does not remove the R/G site, it interacts with the editing effect. Unedited GRIA2Flip is trafficked to dendrites more readily than edited GRIA2Flip (La Via et al., 2013). Editing of the R/G site in GRIA2Flop, but not GRIA2Flip, results in a faster channel closing rate and a faster desensitization rate (Wen et al., 2017). Thus, splicing to the flip variant or leaving the R/G site unedited localizes *Gria2* RNA to the synapses while affecting desensitization kinetics of AMPAR. *Gria2* RNA editing and splicing can change both the RNA and AMPAR localization while modifying channel properties. These two modifications interact, adding another layer to its regulation. With splicing and RNA editing being so common, it is important that we study their interactions, as they may have profound effects throughout the localized transcriptome.

## Alternative Untranslated Regions Drive RNA Localization and Local Translation

Untranslated regions (UTRs) serve important isoform-specific roles by regulating many aspects of mRNA metabolism, including RNA localization, stability, and translation. Utilization of alternate UTRs leads to the incorporation of different *cis-*regulatory sequences, which can be bound by distinct complements of RBPs for differential localization, or microRNAs for translational control. Multiple mechanisms can alter UTRs, such as alternate promoter usage, alternate last exons, and alternative polyadenylation. Here we will discuss examples where varying 5′ and 3′UTRs mediate local gene expression regulation in hippocampal neurites.

Alternative promoters allow for transcription to begin at different first exons and often results in isoforms with different 5′UTRs. In the aged human brain, 60% of genes utilize alternative promoters (Pardo et al., 2013). 5′UTRs can influence various aspects of RNA regulation including localization and translational regulation. For example, transcription of the inducible transcription factor *Npas4* from an upstream promoter results in a variant with a longer 5′UTR that preferentially localizes to hippocampal CA1 dendrites (Brigidi et al., 2019). In the hippocampal slice, excitatory postsynaptic potentials, but not action potentials, lead to selective CA1 dendritic translation of the *Npas4* long 5′UTR variant and its compartment-specific interactor ARNT1. The locally synthesized NPAS1-ARNT1 heterodimers undergo retrograde translocation to the nucleus and activate different sets of genes than when *Npas4* is activated by action potentials. This results in an isoform-specific, UTR-dependent mechanism to decode stimulus-specific synaptic activity into a distinct genomic response (Brigidi et al., 2019).

**Figure 2:**
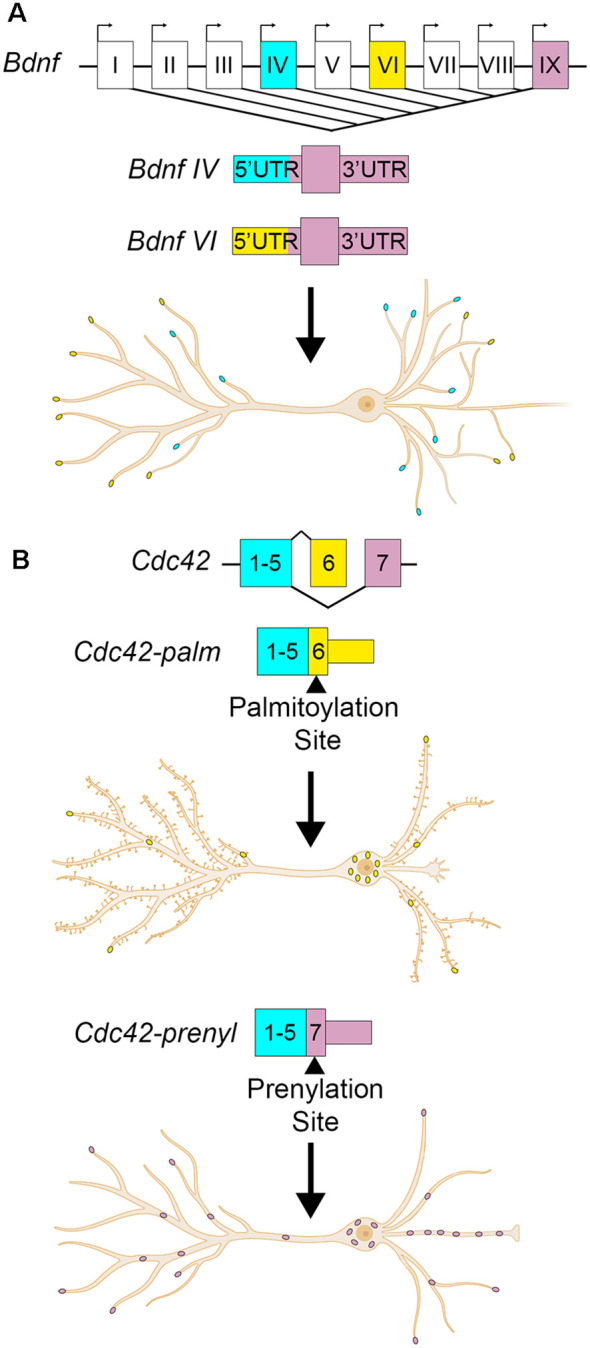
Alternate UTR usage can modify the localization and developmental effects of a transcript. **(A)** *Bdnf* transcription can begin from one of nine alternate promoters. The promoter choice affects the 5′UTR sequence, but not the coding sequence or 3′UTR. *Bdnf* transcribed from the fourth promoter (cyan dots) localizes to proximal dendrites, while transcription from the sixth promoter localizes it to distal dendrites (yellow dots). **Bdnf IV** localization to proximal dendrites promotes dendritic formation. **(B)** *Cdc42* has alternate last exons that determine its 3′UTR. The upstream last exon localizes *Cdc42* mostly to the cell body of the neuron (yellow dots), and contains a palmitoylation site. This 3′UTR variant promotes spine formation. The downstream last exon containing a prenylation site enriches the transcript to neurites and axons (pink dots) and promotes axogenesis.

Brain-derived neurotrophic factor (*Bdnf*) is another example with transcript isoforms that differ only in the UTRs. BDNF is a critical neurotrophin that regulates hippocampal plasticity (Leal et al., 2014). In humans and mice, it has nine alternate promotors and two alternative polyadenylation sites, producing at least 22 transcript variants (Aid et al., 2007; Colliva and Tongiorgi, 2021). Each of the alternate promoters produces an entirely different 5′UTR. The resulting alternate *Bdnf* isoforms have differing roles, regulations, and localizations despite harboring identical amino acid sequences (Metsis et al., 1993; Timmusk et al., 1993; Pruunsild et al., 2007; Foltran and Diaz, 2016). Constitutive and activity-dependent dendritic targeting elements have been identified in the *Bdnf* CDS and 3′UTRs (Oe and Yoneda, 2010; Vicario et al., 2015). *Bdnf* isoform-specific localization is thought to be mediated by permissive or repressive interactions between the 5′UTRs and CDS *cis-* elements (Colliva and Tongiorgi, 2021). Some of the RBPs required for 3′UTR mediated *Bdnf* dendritic targeting have been identified, including CPEB-1 and several ELAV and FXR family proteins (Oe and Yoneda, 2010; Vicario et al., 2015). The RBPs associated with the different 5′UTRs have been bioinformatically identified, but not yet validated (Colliva and Tongiorgi, 2021). Here we will focus on *Bdnf* transcribed from the fourth promoter, also known as promoter IV and previously as the third promoter/promoter III until ~2007 (Zheng et al., 2011). *Bdnf* mRNA transcribed from promoter IV is expressed in an activity-dependent fashion across the rat hippocampus, where it is retained mostly in the soma and proximal dendrites (Chiaruttini et al., 2008; Chapman et al., 2012). The 5′UTR transcribed from the fourth promoter causes the retention of *Bdnf* RNA to the proximal dendrites, whereas the 5′UTRs transcribed from other promoters, including VI, permit localization to distal hippocampal dendrites (Chiaruttini et al., 2008; Baj et al., 2011). Additionally, the alternate 5′UTRs may serve to control the translational efficiency of the RNA. *Bdnf* 5′UTRs repress translation of a reporter construct in rat cultured hippocampal neurons, with promoter IV 5′UTR repressing reporter translation the least, and promoter VI 5′UTR repressing the most (Vanevski and Xu, 2015). *Bdnf* promoter-specific knockout mice show hippocampal cell- and –compartment-specific deficits in dendritic arborization, namely promoter IV knockout mice show decreased apical dendritic arborization in CA1, but not neighboring CA3 (Maynard et al., 2017). Overexpression of BDNF from promoter IV increases the number of primary and secondary dendrites in rat hippocampal neurons at 7 DIV (days *in vitro*) and 18 DIV, respectively (Baj et al., 2011). Collectively, these data support a model in which transcription of *Bdnf* mRNA from promoters IV and VI localize to proximal or distal dendrites, respectively, in a UTR-dependent manner, establishing isoform-specific regulation of different dendritic compartments (Figure 2A; Baj et al., 2011; Colliva and Tongiorgi, 2021).

Activity-dependent transcription at promoter IV is regulated by cAMP response element binding protein 1 (CREB1) and methyl-CpG binding protein 2 (MECP2). Calcium influx upon neuronal activation causes the nuclear translocation of γCAMKII, which leads to phosphorylation of CREB1 (Bito et al., 1996; Deisseroth et al., 1996; Ma et al., 2014; Cohen et al., 2018). CREB1 then binds to the calcium/cyclic AMP response element (CRE) in promoter IV, initiating transcription (Shieh et al., 1998; Tao et al., 1998; Pruunsild et al., 2011). On the other hand, MECP2 can bind to methylated CpG islands in promoter IV, preventing its transcription (Chen et al., 2003; Martinowich et al., 2003; Chang et al., 2006; Zhou et al., 2006; KhorshidAhmad et al., 2016). MECP2 can be phosphorylated in a calcium-dependent manner following neuronal activation, allowing for its dissociation from promoter IV (Chen et al., 2003; Zhou et al., 2006). *Bdnf* at promoter IV is an elegant example of UTR-specific regulation influencing RNA localization. By simply initiating and/or inactivating transcription from a specific promoter, the plasticity at specific synapses can be regulated in a UTR-dependent manner.

Alternate 3′UTRs primarily take two forms- alternate last exons and APA. In the former, the terminal exon is different among isoforms, resulting in completely distinct 3′UTRs. For example, cell division cycle 42 (*Cdc42*) is a Rho-family GTPase that is required for plasticity at CA1 spines (Murakoshi et al., 2011; Kim et al., 2014; Shibata et al., 2021) and has two splice variants with alternate last exons resulting in different C-terminal protein sequences and 3′UTRs (Figure 2B; Munemitsu et al., 1990; Shinjo et al., 1990; Marks and Kwiatkowski, 1996; Olenik et al., 1997). The proximal last exon is known as exon 6 or exon 6B, and is spliced into the brain-exclusive variant CDC42-palm (due to its palmitoylation modification, also known as CDC42-v2, CDC42-E6, CDC42b, and bCDC42, where b stands for brain; Marks and Kwiatkowski, 1996). The distal last exon is termed exon 6A or exon 7, and is spliced into the ubiquitously expressed CDC42-prenyl (due to its prenylation modification, also known as CDC42-v1, CDC42E7, and CDC42u where u stands for ubiquitous; Kang et al., 2008; Roberts et al., 2008). For the rest of this review, CDC42-palm and CDC42-prenyl will be used. *Cdc42-prenyl* is expressed ubiquitously due to its last exon having a stronger 3′ splice acceptor (Yap et al., 2016). In non-neuronal cells, the splicing regulators PTBP1 and PTBP2 repress the inclusion of the *Cdc42-palm* alternate last exon (Yap et al., 2016; Ciolli Mattioli et al., 2019). Downregulation of these factors in early neuron development promotes the inclusion of the *Cdc42-palm* alternate last exon with the weaker 3′splice acceptor (Zheng et al., 2012; Yap et al., 2016). While the RNAs of each isoform localize to both neurites and soma, *Cdc42-prenyl* has higher expression than *Cdc42-palm* in cultured neurites (Taliaferro et al., 2016; Ciolli Mattioli et al., 2019; Lee et al., 2021) and hippocampal neuropil (Farris et al., 2019). *Cdc42-prenyl* mRNA has a longer 3′UTR that mediates its localization to neurites in mouse cortical neurons and mouse embryonic stem cell-derived neurons, where it is locally translated (Marks and Kwiatkowski, 1996; Ciolli Mattioli et al., 2019). CDC42-prenyl promotes axogenesis in hippocampal neurons (Yap et al., 2016). While the role of CDC42-prenyl prenylation in neurons is not yet understood, it may anchor the protein to axons since this modification allows its association with the membrane in *S. cerevisiae* (Estravís et al., 2017). Palmitoylation of CDC42-palm attaches the fatty acid palmitate to either Cys188 or Cys189, which is required for induction of dendritic filopodia, and the development of long protrusions in cultured neurons (Wirth et al., 2013). Palmitoylation of CDC42-palm also stabilizes hippocampal spines and rescues spine density in 22q11DS transgenic mouse models (Moutin et al., 2017). Glutamate treatment of hippocampal cultures induces a rapid depalmitoylaion of CDC42-palm and its dispersal from spines (Kang et al., 2008). Thus, the alternate 3′UTRs mediate the isoform-dependent localization of *Cdc42* in hippocampal neurons, while posttranslational modifications can alter their activity and function.

Another mode of alternative 3′UTR generation is alternative polyadenylation, which results from transcription termination at different points along the same terminal exon, resulting in shortened or elongated versions of the 3′UTR. 79% of mouse coding genes have alternative polyadenylation sites (Hoque et al., 2013). APA is especially prevalent in the brain, where tissue-specific 3′UTR lengthening is enriched (Miura et al., 2013). In excitatory neuron-differentiated mESCs, 4,149 genes were found to have differentially localized alternate 3′UTRs of which 3,675 corresponded to APA isoforms, and 474 to alternate last exons (Ciolli Mattioli et al., 2019). While the specific mechanisms governing preferential localization of alternate UTR isoforms to specific compartments remains unknown for the majority of transcripts, compartment localized RNAs in the adult hippocampus have longer 3′UTRs that are more stable and contain more microRNA seed sequences and RBP motifs compared with non-localized RNAs (Tushev et al., 2018), providing greater opportunity for regulation. Indeed, neurite-enriched 3′UTRs contain binding motifs for localization-linked RBPs such as muscleblind-like (MBNL) family RNA-binding proteins (Taliaferro et al., 2016; Tushev et al., 2018), zip-code-binding protein (ZBP1; Tushev et al., 2018), and fragile X mental retardation protein (FMRP; Tushev et al., 2018). Longer 3′UTRs allow for more microRNAs to bind and repress translation (Legendre et al., 2006; Hu and Li, 2017; Epple et al., 2021). There is evidence that up to 88% of mouse hippocampal neurite mRNAs are targeted by synaptic microRNAs (Epple et al., 2021), suggesting that noncoding RNAs have an underappreciated role in localized gene expression (Hu and Li, 2017; Epple et al., 2021).

**Figure 3:**
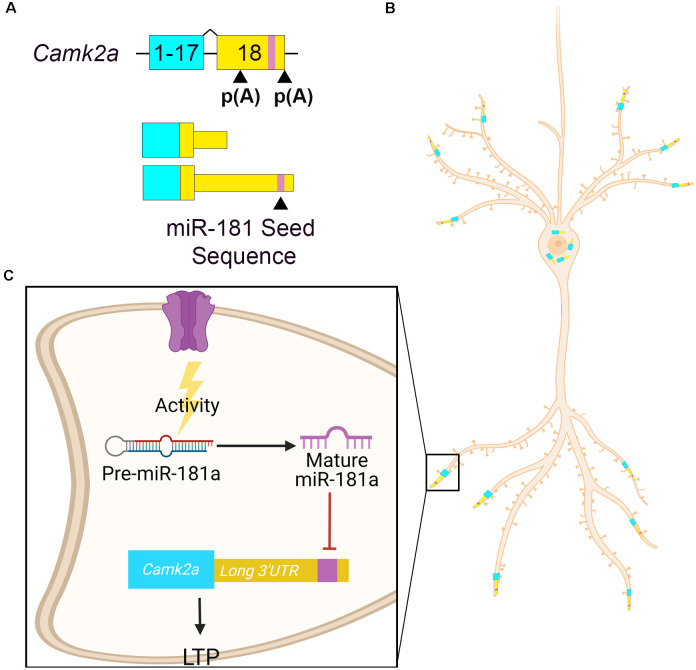
Camk2a APA drives its localization and activity-dependent miRNA regulation. **(A)** *Camk2a* APA [p(A)] in exon 18 determines the 3′UTR length. The distal p(A) site results in a longer 3′UTR that contains a miR-181a binding site (purple). **(B)** *Camk2a* with the shorter 3′UTR localizes to the cell soma, whereas *Camk2a* with the longer 3′UTR localizes to neurites. **(C)** Pre-miR-181a is present at hippocampal synapses. In response to synaptic activity, it is processed by DICER into its mature active form. Mature miR-181a then represses the translation of *Camk2a* by binding to its seed sequence in the long 3′UTR. This results in the inhibition of long-term potentiation (LTP). Expressing the longer 3′UTR increases *Camk2a’s* regulatory potential.

Calcium calmodulin-dependent protein kinase II alpha (*Camk2a)* is a well-characterized gene involved in memory formation that encodes alterative isoforms with different length 3′UTRs due to alternative polyadenylation (Figure 3A). *Camk2a* mRNA containing the longer 3′UTR is enriched in hippocampal neuropil (Tushev et al., 2018). *Camk2a* dendritic localization is dependent on elements within its 3′UTR (Mayford et al., 1996; Mori et al., 2000) as transgenic reporter mice containing only the *Camk2a* 3′UTR is sufficient to localize a lacZ transcript to hippocampal dendrites (Mayford et al., 1996). miR-181a is a locally processed microRNA that controls hippocampal plasticity *via* alternative 3′UTR-mediated translational repression (Sambandan et al., 2017). miR-181a is present in rodent hippocampal dendrites and synaptosomes (Epple et al., 2021). miR-181a is present in rat CA1 dendrites in its pre-miR form and is processed locally to its mature form by DICER upon synaptic activation (Sambandan et al., 2017). After maturation, miR-181a inhibits the local translation of *Camk2a*, which is required for memory formation (Miller et al., 2002; Sambandan et al., 2017). A GFP reporter construct containing the *Camk2a* long 3′UTR showed decreased activity-dependent translation in hippocampal dendrites compared to a construct containing the short *Camk2a* 3′UTR lacking the miR-181a seed sequence (Sambandan et al., 2017). Thus, the inclusion of miR-181a seed sequence in *Camk2a* long 3′UTR allows for local translational repression at active synapses (Figures 3B,C). miR-181a also downregulates the expression of rat hippocampal *Gria2* mRNA (Saba et al., 2012). miR-181a expression in the rat dorsal hippocampus increases after learning tasks and is associated with inhibition of protein kinase AMP-activated catalytic subunit alpha 1 (*Prkaa1*) RNA, the catalytic subunit of AMP-activated protein kinase (AMPK), a regulator of plasticity (Potter et al., 2010; Zhang et al., 2017). Thus, local activity-dependent pre-miR-181a processing leads to the repression of several synaptic transcripts that are essential to plasticity.

miR-181a demonstrates how isoform expression can interact with microRNA-mediated local translational repression through alternative 3′UTRs. In fact, 43.3% of predicted microRNA target sites in human 3′UTRs are found in alternative UTR segments (Majoros and Ohler, 2007). Furthermore, miR-181a is predicted to target a significant number of 3′UTRs which undergo alternative splicing (Wu et al., 2013). By including a longer 3′UTR, RNAs can be localized to neurites more efficiently, possibly through additional RBP binding or greater RNA stability (Tushev et al., 2018), where they can be regulated by microRNAs in an activity-dependent manner.

## Discussion

Each step of post-transcriptional regulation from RNA localization to stability to translation and degradation can be altered by changing the *cis* RNA regulatory sequences in a given transcript. In morphologically complex cells like neurons, alternative isoform expression is used to tune these regulatory processes and provide distinct subcellular compartments with the necessary transcripts to support specialized functions. Unraveling this code is vital to fully understanding how RNA is regulated across different contexts and cell types to support neuronal functions. The genes presented in this review illustrate how alternate isoform expression mediates localized RNA regulation to ensure proper hippocampal neuron development and plasticity.

## Are There Subregion-Specific Splicing Programs in The Hippocampus?

Isoform-specific regulation of *Pex5l* and *Dlg3* leads to the proper expression and localization of HCN1 and NMDAR subunits, respectively, which are vital for the development of proper electrical activity for synaptic maturation (Ewald and Cline, 2009; Stoenica et al., 2013). *Pex5l* exon 1a expression is highest in CA1, consistent with the critical role of HCN1 in CA1 pyramidal cells (Magee, 1999; Piskorowski et al., 2011). However, it remains unknown how subregion-specific splicing programs in the hippocampus are implemented and thus, further investigation is needed. The hippocampus has cell- and compartment-specific transcriptomes, as it is divided into 4 major subregions (CA1, CA2, CA3, and the dentate gyrus) with diverse gene expression schemes to control subregion-specific properties and functions (Masser et al., 2014; Cembrowski et al., 2016; Farris et al., 2019). Several studies have identified alternative splicing programs that readily distinguish neuron cell classes (glutamatergic, GABAergic, glia; Zhang et al., 2014; Furlanis et al., 2019; Sapkota et al., 2019; Feng et al., 2021; Joglekar et al., 2021) and to a lesser extent distinguish neuron subclasses (CA1, CA3; Furlanis et al., 2019; Joglekar et al., 2021), indicating that alternate isoform expression is a driver of functional specification (Furlanis et al., 2019). Cell-specific expression of transcription factors and epigenetic modifiers likely induce expression of these specialized transcriptomes, but it is still unknown how they communicate with the splicing machinery to induce expression of one isoform over another. Cell-specific transcriptomes must further change at specific developmental windows (Weyn-Vanhentenryck et al., 2018) and also be actively maintained into adulthood (McCann et al., 2021). There are likely many players involved, as neuronal splicing regulatory networks require combinatorial interactions between splicing factors and RBPs (Raj and Blencowe, 2015; Vuong et al., 2016). The identification of these players using cell- and isoform-specific profiling approaches in combination with gene knockout methods will shed light on the cell- and context-dependent interplay between spliceosome components, transcription factors, and RBPs.

## What Regulatory Elements Influence Localized Isoform Expression?

As evidenced by *Npas4, Bdnf, Cdc42*, and *Camk2a*, both 5′ and 3′UTRs can direct the trafficking of transcripts to hippocampal dendrites. The inclusion of different *cis* sequences in the UTRs increases the capacity for regulating RNA characteristics, such as localization and stability. With alternative promoters, alternative polyadenylation, and alternative last exons being so pervasive in neurons (Pardo et al., 2013; Tushev et al., 2018), this leads to an underappreciated amount of diversity in UTR-dependent regulation. The functional impact of this UTR diversity on local RNA regulation is still largely unknown. The regulation and trafficking of UTR-dependent RNA localization is mediated by RNA-binding proteins (Miura et al., 2013; Mayya and Duchaine, 2019; Bae and Miura, 2020), but for the overwhelming majority of RNAs, the binding partners and mechanisms that mediate localization along with the contexts under which it happens are still being discovered. Indeed, hippocampal compartment-specific sequencing studies have identified thousands of synaptically localized RNAs that display cell-type specificity (Cajigas et al., 2012; Farris et al., 2019), hundreds of which have alternate UTRs (Tushev et al., 2018), underscoring the diversity and complexity of RNA regulation occurring in dendrites. While RBP motifs and dendritic targeting elements in specific transcripts have been characterized, it remains unknown how combinations of *cis* sequences work in concert to induce specific patterns of localization. and how different contexts or dynamic systems expressing distinct complements of RNAs and RBPs affect these interactions. The tools in this regard have improved over time to include *in silico* motif predictions to pair with cross-linking immunoprecipitation (CLIP) data in order to identify the locations of RBPs along target RNA transcripts. Cell-specific RNA-RBP prediction networks that modulate local transcriptomes can be generated by combining compartment-specific transcriptome data with RBP-CLIP data, or when technically feasible, performing compartment-specific RBP-CLIP in different contexts. Fully elucidating UTR-mediated local RNA regulation by unraveling the RNA localization code may be beyond our technological reach at this time. However, advances in highly-multiplexed and iterative single-molecule imaging techniques to map RBP-RNA interactions *in situ* provide much optimism.

RNA editing is another tool that can regulate the localization of plasticity-related transcripts. By interacting with splicing, RNA editing provides an additional regulatory layer through which transcripts can be modified in response to activity. Of the 17,831 known RNA editing sites in the mouse genome, 682 have been detected in the hippocampus (Stilling et al., 2014). It is clear that editing plays a role in the hippocampus, but its relationship with splicing still requires further study. We have seen an example of splicing-editing interaction in *Gria2*, but the exact molecular players remain unidentified. RNA editing can occur co-transcriptionally like splicing, and some RNA editing enzymes have been implicated in splicing (Kapoor et al., 2020). It will be interesting to determine whether RNA editing proteins and splicing factors co-localize to coordinate editing with splicing.

microRNA regulation is another way that splicing can impact local RNA expression. Alternate UTRs increase the regulatory potential of microRNAs. By utilizing 3′UTRs containing a different mix of seed sequences, microRNA-mediated regulation can be adjusted to meet specific regulatory needs. Activity is a key signaling step in the maturation process of miR-181a, allowing it to be loaded onto the RNA-induced silencing complex (RISC) to repress translation of target transcripts such as the long 3′UTR neurite-localized *Camk2a*. How the RISC identifies miR-181a and how it recruits target transcripts remains unknown. Indeed, the prevalence and mechanisms of microRNA-mediated control of localized RNA need further elucidation, as it is a potentially widespread method of local gene expression regulation. Understanding the full extent of this regulatory mechanism may be key to understanding the role of local translation and its role in synaptic plasticity.

It remains an open question as to how alternate isoforms impact local translational capacity. Genome-wide measures of isoform-specific transcription and translation in mature neuronal cultures from differentiated human embryonic stem cells demonstrate that regulatory sequences in long 5′ and 3′UTRs generally repress translation, with the strongest effect from 3′UTRs (Blair et al., 2017). This is consistent with the fact that localized RNAs have longer UTRs and require translational repression for transport. Structural features detected in some dendritic transcripts, such as high G-C content in 5′UTRs and upstream open reading frames, are also consistent with translational repression (Falley et al., 2009). Low translational capacity is often equated with monosome association and a recent study showed that localized transcripts in CA1 exhibit a preference for monosome translation (Biever et al., 2020). The monosome preferring transcripts encoded a full range of low- to high-abundance proteins in the neuropil, suggesting that monosomes are an essential source of synaptic proteins (Biever et al., 2020). As technologies continue to advance for lower input samples, it will be critically important to directly test the impact of alternate isoforms on local translational capacity.

## What Other Emerging Forms of Splicing Warrant Further Study?

Several other forms of splicing require further study to understand their roles in local RNA regulation as it relates to hippocampal neuron function. Circular RNAs (circRNAs) are non-linear transcripts without a distinct 3′ or 5′ end that are produced as a result of back-splicing (Gokool et al., 2020). In the hippocampus, circRNAs derived from synapse-related genes such as *Homer1*, *Elavl*, and *Nlgn1* are present at hippocampal synapses (You et al., 2015). The linear mRNA for Staufen2 (*Stau2*) is almost exclusively present in the cytoplasm, while circStau2 localizes to synapses in both mouse and human neurons, suggesting a synaptic role for some circRNAs (Rybak-Wolf et al., 2015). Retained introns are another form of splicing in which introns are included in the mRNA. Retained introns can contain *cis*- elements such as constitutive transport elements (CTE) that are bound by RBPs (Li et al., 2006, 2016). Staufen2 protein has been shown to localize *Calm3* mRNA to hippocampal dendrites in a retained-intron-3′UTR-dependent manner (Sharangdhar et al., 2017). NXF1 regulates the nuclear export of its own mRNA with a retained intron, leading to its protein isoform colocalizing in RNA granules with Staufen2 (Li et al., 2016). Microexons are 3–27 nucleotide long sequences that insert small stretches of in-frame amino acids into protein products (Gonatopoulos-Pournatzis and Blencowe, 2020). In the mouse hippocampus, differential splicing leads to significant changes in protein isoform expression, and microexons are enriched in alternative splicing events that lead to proteome changes in neurons (Reixachs-Solé et al., 2020). Microexons programs are highly conserved and are involved in critical neuronal functions such as neurogenesis, vesicle trafficking, and RNA binding proteins (Gonatopoulos-Pournatzis and Blencowe, 2020). The disruption of neuronal microexons is associated with autism spectrum disorders. RNAseq on postmortem brains showed that 30% of detected microexons were significantly different between autistic and control brains (Irimia et al., 2014). Further work will be needed to reveal the cell- and compartment-specific role for these mechanisms in regulating localized gene expression.

## Conclusion

Developmental and activity-regulated gene expression programs can utilize cell- and context-specific RNA codes necessary to support compartment-specific functions. Despite all the elegant work described in this review, we have only scratched the surface of transcript isoform-dependent local RNA regulation. Recent work has cataloged cell- and compartment-specific isoform expression, but many questions remain with regards to the functional roles that specific sequences and their interactors play in regulating local transcriptomes. Advances in long-read technologies, such as nanopore sequencing, have illuminated the extent of previously underestimated levels of RNA isoform diversity. Nanopore sequencing allows for the direct sequencing of full-length transcripts, instead of the shorter reads utilized by RNAseq. This technology was able to identify 33,984 isoforms from 10,793 genes in human cells, of which 52.6% had an unannotated splice junction (Workman et al., 2019). This technology can be used to analyze isoforms in different cellular compartments, as nanopore sequencing across 13 cell lines revealed that there are major differences in isoforms expressed in the nucleus vs. cytoplasm, including intron-retention enrichment in nuclear transcripts (Zeng and Hamada, 2020). Moving forward, insights revealed from sequencing methodologies such as long read (Bolisetty et al., 2015; Deamer et al., 2016) and 5′/3′ end sequencing need to be integrated with functional assays and spatially resolved single-molecule imaging techniques to fully unravel the ways in which the RNA code impacts local RNA regulation in specific contexts. The hippocampus remains a useful model system to uncover splicing-dependent regulation of localized transcriptomes to impact neuron function.

## Author Contributions

All authors contributed to the article and approved the submitted version.

## Conflict of Interest

The authors declare that the research was conducted in the absence of any commercial or financial relationships that could be construed as a potential conflict of interest.
